# Report of the Necrologist

**Published:** 1873-07-15

**Authors:** 


### REPORT OF THE NECROLOGIST.

CHICAGO, March 13th, 1873.

TO THE PRESIDENT AND MEMBERS OF THE ALUMNI ASSOCIATION OF THE CHICAGO MEDICAL COLLEGE :

*Gentlemen:* — The Necrologist has the honor to report the completion of biographical sketches of Drs. M. N. Rust and G. W. Willson, which are here presented. He is anxious to gain information regarding Drs. D. J. Allaban, J. M. Kendall, E. B. Rockwell, J. J. Samuels, and Lucius Dillie, all deceased alumni of our alma mater. He feels certain that no alumnus who shall become aware of this fact will fail to report to him any facts he may be in possession of regarding the history of any of these gentlemen.

Respecfully submitted,

NORMAN BRIDGE, *Necrologist*,
267 W. Monroe street.

### GEORGE WARE WILLSON, CLASS OF 1864.

Dr. Willson was the only child of James L. and Clarissa W. Willson, and was born in Tremont, Tazewell County, Illinois, December 10th, 1838. His father has been much of his life a druggist. His mother's maiden name was Clarissa Richmond Ware. His parents removed to Chicago, in 1846. On the opening of the High School he entered it as a pupil, and graduated with the first graduating class. He then spent some time in a drug store of his father's, and afterward began the study of medicine with Drs. Andrews and Johnson. He attended lectures in the Chicago Medical College, and graduated with the class of 1864. On the breaking out of the war, young Willson entered the 12th Illinois Infantry. Subsequently he was in the hospital under Dr. Horace Wardner, of Cairo, following the army in various marches. He resolved, in the autumn of 1862, to enter, if possible, the medical service of the navy, and accordingly presented himself for examination at Philadelphia, September 25th, 1862. He passed successfully his examination, and was at once appointed Acting Assistant Surgeon. An order was issued to him on the next day to report for duty to Rear Admiral Lee. He was assigned to duty at Newberne, N. C., and for a time had medical charge of the Steamer "Hetzel." Afterwards he had charge of the hospital at Newberne, and was on duty in both at the time of his death, which occurred on September 24th, 1864.

His work was very hard, with the double labor of his two positions, and his health gradually failed, until he should have been relieved from duty. But during those years there were no surgeons unemployed; it was difficult for a useful man to be spared, and Willson remained at his post.

There was sent, during the latter part of the summer of 1864, from the far South, a quantity of clothing that had been taken from hospitals where the yellow fever was prevailing. Some of this was sent to the hospital at Newberne.

Yellow fever soon broke out among Willson's patients, and after battling against it in the bodies of others, he himself was attacked with it, and died September 24th. His age was 24 years, 9 months and 14 days.

Dr. Willson had a great love for his profession. He had descended from a long line of physicians on his mother's side. His great great grandfather, William Ware, was a surgeon in Wales, and emigrated in the early time to this country. His great grandfather and grandfather were both physicians, and both named George Ware.

Willson was fond of all scientific studies; he had a good inventive genius, and took great interest in machinery. His disposition was mild and cheerful; his opinions were founded in reason, and not on tradition; and he had a hatred of cant and ceremony. In religion he was of the Unitarian belief, and belonged to that denomination. He was educated in this religion, and his reason and conscience approved of it. He was a strict temperance man, and took the pledge, when only eight years old, of Father Matthew. He was a strong anti-slavery man, and detested oppression anywhere, and of any sort. He was never married. An old classmate of his, a man of candor, and who was his warm friend — Dr. S. H. Bottomly — puts on record the following estimate of the character of Dr. Willson, as well as some facts regarding his life: "It was my good fortune to know him quite intimately, from the time he commenced his studies until he entered the naval service. We occupied the same room for nearly a year, during which time we pursued the same studies. Willson was an average student; did not acquire knowledge easily, nor comprehend readily; but ideas once obtained, remained fixed in his mind; while, by industry and constant application, he overcame difficulties which otherwise would have been unsurmountable. He was of an exceedingly genial disposition; was easily excited to laughter, which, when once heard, was never forgotten. He was slow to anger, having his passions well under control. Although of an unassuming nature, he kept his eyes open to the chances in life; and although young, stood high in the regards of his friends."

---

MELVILLE NIEL RUST, CLASS OF 1865.

The subject of this sketch, was the son of Elam and Mary P. Rust, and was born at Waddington, New York, November 10th, 1840. His father was a native of Vermont, the Rust family having removed from Connecticut in 1760, and settled in Windsor county. His mother's name was Foot, and was born in New York. She is still living. Her father was the first pioneer of St. Lawrence county, having moved from Vermont.

Elam Rust was a lawyer, and practiced in St. Lawrence county until 1842, when he removed to St. Clair county, Illinois. He died at Macon in 1857. He was, during most of his residence in Illinois, publisher of a newspaper. Dr. Rust had two brothers and one sister, all of whom are now living.

Dr. Rust's educational advantages were chiefly the common school, which, in that day and region, were not of the best. After the death of his father, he was placed for nearly two years at a high school at Ogdensburg, New York. Leaving there in the spring of 1859, young Rust at first, seemingly by association, drifted into the printing business, and, until he finally engaged in the study of medicine, he was, at irregular intervals, engaged in this employment. During the latter part of 1859 he was in a printing office in Omaha, Nebraska. Then he entered a drug store, where he continued for some time. In 1861 he went to St. Louis, whither his mother had removed. He soon accepted a position as manager of a printing office in Effingham, Illinois. He paid a visit to Cairo, to his acquaintances among the recruits just then congregating there, and, among other troops, he found the 8th Illinois Infantry, commanded by Col. Oglesby, now Senator. This regiment had many old friends, and he enlisted in Co. B, on July 25th, as a private. Soon after, the 12th Illinois Infantry, under Col. John McArthur, reached Cairo, and Dr. Horace Wardner — once a professor in the Chicago Medical College — was its Surgeon. An acquaintance of the Rust family, he requested that Melville be transferred to his regiment, and be detailed as his Hospital Steward. This request was granted. In the capacity of Hospital Steward, young Rust served at Fort Donnelson and Shiloh. It was while thus acting that he conceived the notion of studying medicine. He began his study at once, and Dr. Wardner gave him every advantage. At this time, the army around Corinth was deficient in regular medical officers, and "Contract Surgeons," were, to a large degree, on duty with the regiments. Mr. Rust, before long, applied for a discharge from the service, that he might accept a position as "Contract Surgeon," believing that, should his request be granted, he would always be on duty under the direction of an experienced surgeon. He obtained his position, and was assigned to the 81st Ohio Infantry, which, at that time, had no regular medical officer. So well pleased with his conduct were the officers of the regiment, that they obtained the office for him, from the Governor of Ohio, of a commission as Assistant Surgeon. Rust had never attended a course of lectures, and here was a "short cut" to a professional position. He declined the offer. Meanwhile, large bodies of troops were gathering around Vicksburg, and men were rapidly getting sick, and medical officers were deficient. All the contract surgeons near Corinth were ordered to the front, Rust among them, being assigned to a Wisconsin regiment. This regiment occupied an isolated position, and had no other medical officer of any description, and had several hundred sick men. He procured transportation, and sent such as could be moved to Memphis to a general hospital. Without counsel of experienced physicians, there were many of the cases for which, with his meager knowledge he could do nothing. Impressed with his awful responsibility, he often afterwards related, with tears, the anguish at the duty that was placed upon him, and he incompetent to discharge it; and on the arrival of a surgeon from Wisconsin, he secured a cancellation of his contract.

He now became Quartermaster's Clerk, under Capt. B. J. T. Hanna, and remained until after the fall of Vicksburg, when he resigned and came north. In the fall of 1863 he entered the Chicago Medical College, and at the end of the term went to Mound City, and spent the season under the tutelage of Dr. Wardner, who had charge of a hospital at that place. He returned to the lecture term of 1864 – 5, and graduated at the close of the season. He now applied to Gov. Oglesby for a position as Surgeon in one of the new regiments then forming. Oglesby wrote in reply that, on account of his youth, he could only apply for a place as Assistant Surgeon, and enclosed a permit for him to appear before the State Examining Board for examination for such a position. By some mistake, the paper was so worded as to allow him to apply for either a position as Surgeon or Assistant Surgeon, and he at once presented himself to Dr. Daniel Bramont, the member of the Examining Board for Chicago, for examination for the higher position. Dr. Bramont was reluctant to admitting one so young in years and in the profession to examination for Surgeon, but was willing to examine him for Assistant Surgeon. But Rust insisted that the issue ought to depend upon the merits of the examination itself, and on nothing else, and Bramont consented.

The examination was long and severe, and at its close, Rust received the certificate for which he applied. He was at once appointed Surgeon, and assigned to the 154th Illinois Infantry, and was, with his regiment, ordered to Nashville. Rust was soon detailed to join the staff of the Post Commander, and take charge of the Post Hospital. Here he remained until his death, in the summer of the same year. The 154th regiment was stationed near Nashville, and his Colonel having died early in August, Dr. Rust rode out to attend his funeral. His horse was high-spirited and restive, and became unmanagable, and plunging down one of the narrow streets of the town, bore his rider into the midst of a wagon-train which blocked up the way. Rust was injured, and soon dismounted and gave his horse to an Orderly, and retiring to an adjacent office, asked permission to lie down. After rest he felt better. During the night he was attacked with dysentery. He continued, however, to prescribe for others, until the Hospital Steward, satisfied his mental faculties were becoming impaired, sent for Dr. Perkins, Medical Director. Dr. P. declared Rust beyond the reach of treatment, and he died August 13th. His body was embalmed and brought to Chicago and buried in Rose Hill Cemetery.

In physique Dr. Rush was erect, strong and robust. He was a man of great resolution, and conclusions once formed were acted upon promptly and vigorously. With a pleasing address and genuine nature, he had many friends whom he would do almost anything to serve. He had an exalted idea of the honor of the profession, and his industry and enthusiasm marked him as one who, had he been spared, would have earned substantial honors.

In a communication to the writer, Dr. Wardner says of him, speaking of his first connection with the army: "He entered the 12th regiment in the summer of 1861, and turning his attention to the study of medicine, made such progress and showed so much ability and judgment that he was discharged in the summer of 1862, for the purpose of accepting an appointment as Acting Assistant Surgeon." In referring to his character, Dr. Wardner says: "His was a noble, generous nature. He was unusually ready in acquiring knowledge, and his judgment in the application of his knowledge to the every-day work of life was superior."

His devotion to his widowed mother was worthy of imitation.

